# Differential Release of β-Casomorphins from A1 and A2 Milk During Standardized Gastrointestinal Digestion Quantified by CE–MS

**DOI:** 10.3390/foods15101776

**Published:** 2026-05-18

**Authors:** Tahereh Tehrani, Laura Pont, María Vergara-Barberán, Bibiana Juan, Antonio José Trujillo, Fernando Benavente

**Affiliations:** 1 Department of Chemical Engineering and Analytical Chemistry, Institute for Research on Nutrition and Food Safety (INSA·UB), University of Barcelona, 08028 Barcelona, Spain; ttehrani@ub.edu (T.T.); laura.pont@ub.edu (L.P.); maria.vergara@uv.es (M.V.-B.); 2 Serra Húnter Program, Generalitat de Catalunya, 08007 Barcelona, Spain; 3 Department of Analytical Chemistry, University of Valencia, 46100 Burjassot, Spain; 4 Center for Innovation, Research, and Transfer in Food Technology (CIRTTA), Department of Animal and Food Science, Faculty of Veterinary Medicine, Autonomous University of Barcelona, 08193 Bellaterra, Spain; bibiana.juan@uab.cat (B.J.); toni.trujillo@uab.cat (A.J.T.)

**Keywords:** β-casomorphins, bovine milk, CE-MS, INFOGEST, sample preparation, solid-phase extraction

## Abstract

β-Casein A1 and A2 (β-CN-A1 and β-CN-A2) are the two predominant β-CN proteoforms in bovine milk. β-CN-A1 has been associated with a greater propensity to release opioid peptides, such as β-casomorphin-7 (β-CM-7) and β-casomorphin-5 (β-CM-5), during gastrointestinal (GI) digestion, which may have adverse biological effects. This has stimulated growing interest in milk from cows carrying the β-CN A2A2 genotype (A2 milk), which requires reliable characterization methods. In this work, we developed a rapid, selective, and sensitive capillary electrophoresis–mass spectrometry (CE-MS) method for the accurate identification and quantification of β-CM-7 and β-CM-5 in milk hydrolysates from in vitro GI digestion of bovine milk. The method showed good linearity (R^2^ > 0.99, over 0.5–100 mg/L for β-CM-7 and 0.25–100 mg/L for β-CM-5), limits of detection (0.25 and 0.10 mg/L), and repeatability (<0.2% for times and <1.4% for areas), and tandem mass spectrometry (MS/MS) allowed confirmation. The method was applied to A1A1 and A2A2 milk digested using the standardized INFOGEST protocol, followed by solid-phase extraction. β-CM-7 was detected and quantified only in A1A1 digests (0.98 mg/L), whereas β-CM-5 was not detected (<0.10 mg/L). These results indicate a differential release of β-CMs from A1 and A2 milk and support the method’s suitability for β-CM profiling, which may help assess A2 milk quality control and β-CM health impact.

## 1. Introduction

Bovine milk is a major dietary source of protein, comprising approximately 80% caseins and 20% whey proteins [1,2]. β-Casein A1 and A2 (β-CN-A1 and β-CN-A2), which differ by a single amino acid at position 67 (histidine in A1 and proline in A2), are the two predominant β-CN proteoforms in bovine milk. Over the years, these proteoforms have attracted increasing scientific interest due to their distinct health implications [3,4,5,6,7,8]. These effects are primarily associated with β-CN-A1, which shows a greater propensity to release opioid peptides, such as β-casomorphin-7 (β-CM-7) and β-casomorphin-5 (β-CM-5), during gastrointestinal (GI) digestion [3]. These peptides have been proposed to exert opioid-like bioactivity and to modulate gastrointestinal and inflammatory responses associated with various human health conditions, including alterations in GI function, gut inflammation, type-1 diabetes mellitus, cardiovascular diseases, and neurological disorders [5,6,7,8]. Despite limited human clinical evidence supporting these potential adverse effects, they have nonetheless boosted interest in milk from cows carrying the β-CN A2A2 genotype, commonly referred to as A2 milk, which contains exclusively the β-CN-A2 proteoform and is often regarded as a healthier alternative [9]. However, the health impacts of released β-CMs remain a topic of ongoing debate, with studies reporting inconsistent outcomes [3,4]. Individual variability and methodological differences, including the digestion protocol employed, likely contribute to the complexity of the findings [10,11,12,13,14]. Additionally, controversy persists over whether β-CN-A2 can release small amounts of β-CMs and over performance of the currently available analytical methods [3,4]. In light of these factors, there is a growing need for reliable analytical methods, which in combination with standardized digestion protocols [15], can accurately identify and quantify β-CMs in digested milk and dairy products. Indeed, both the occurrence and concentration of β-CMs in these products may vary substantially due to differences in proteolytic enzyme activity and processing conditions [10,11,12,13,14]. Standardized digestion protocols, such as the widely adopted INFOGEST, provide robust frameworks for static in vitro simulations of GI food digestion, enabling consistent and reproducible analysis of digestion-derived hydrolysates [15]. Such protocols are currently critical for systematically investigating potential difference in β-CM release from β-CN-A1 and β-CN-A2.

Regarding the analytical methods, immunoassays and liquid chromatography–mass spectrometry and -tandem mass spectrometry (LC-MS and LC-MS/MS) have long been regarded as the gold standard for β-CM analysis in milk and dairy products [12,13,14,16,17,18,19]. However, these techniques face limitations when dealing with complex matrices and low-abundance peptides, especially immunoassays, which lack molecular mass confirmation [17]. The development of capillary electrophoresis–mass spectrometry (CE-MS) methods for milk protein analysis [20], has opened new avenues for β-CM determination. CE-MS offers several advantages, including low consumption of samples, reagents, and solvents, high-efficiency separations, short analysis times, and complementary selectivity for ionizable molecules such as peptides [21,22,23,24], making it particularly suitable for the analysis of food components [25,26,27], including bioactive peptides such as β-CMs in hypoallergenic milk hydrolysates [28].

In this study, we developed a rapid, selective, and sensitive CE-MS method for the accurate identification and quantification of β-CM-7 and β-CM-5 in bovine milk hydrolysates obtained using the standardized INFOGEST protocol. The method was validated using peptide standards in terms of linearity, limit of detection (LOD), limit of quantification (LOQ), and repeatability. Additionally, capillary electrophoresis–tandem mass spectrometry (CE-MS/MS) was employed to further confirm peptide identity through their characteristic MS/MS fragmentation patterns. β-CMs in A1A1 and A2A2 milk hydrolysates were then analyzed following clean-up and preconcentration using solid-phase extraction (SPE) microcartridges. The established method enables accurate identification and quantification of low levels of β-CMs in milk hydrolysates from GI digestion, providing a reliable analytical platform for β-CM profiling. This approach proved useful for assessing the differential release of β-CMs from A1 and A2 milk during standardized GI digestion.

## 2. Materials and Methods

### 2.1. Chemicals and Reagents

All chemicals used in the preparation of buffers and solutions were of analytical reagent grade or better. Sodium hydroxide (NaOH, ≥99.0%, pellets), propan-2-ol (≥99.9%), trifluoroacetic acid (TFA, ≥99.0%), acetic acid (HAc, glacial), formic acid (FA, 98.0%), acetonitrile (ACN, LC and LC-MS grade), calcium chloride dihydrate (CaCl_2_·2H_2_O, ≥99.0%), bile salts, and water (LC-MS grade) were purchased from Merck (Darmstadt, Germany). α-Amylase from human saliva (≥1500 U/mg protein), pepsin from porcine gastric mucosa (≥250 U/mg protein), and pancreatin from porcine pancreas (8× USP specifications), used for the INFOGEST digestion protocol, were also supplied by Merck. β-Casomorphin-5 (β-CM-5, Tyr-Pro-Phe-Pro-Gly, M_r_ 579.2693) was obtained from Merck, while β-casomorphin-7 (β-CM-7, Tyr-Pro-Phe-Pro-Gly-Pro-Ile, M_r_ 789.4061) was supplied by Bachem (Bubendorf, Switzerland).

### 2.2. Apparatus and Procedures

Centrifugation at controlled temperature (35 °C) was carried out using a Micro 220 centrifuge (Hettich Zentrifugen, Tuttlingen, Germany). Background electrolyte (BGE) pH was measured with a pHmeter Sension+ PH3 (Hach, Ames, IA, USA), while solvent evaporation from samples was made with a SpeedVacTM concentrator (Thermo Fisher Scientific, Waltham, MA, USA). SPE was carried out using an Oasis hydrophilic–lipophilic balance (HLB) 96-well micro-elution plate (30 µm, 2 mg) (Waters Corp., Milford, MA, USA). Agitation was performed with a Vortex Genius 3 (Ika^®^, Staufen, Germany).

#### 2.2.1. Sample Preparation

Milk samples from three individual cows carrying the β-CN A1A1 and A2A2 genotypes were obtained through the Center for Innovation, Research, and Transfer in Food Technology (CIRTTA, Autonomous University of Barcelona, UAB). Genetic information of individual cows was provided by CONAFE (Confederation of Spanish Friesian Associations, Valdemoro, Madrid, Spain). Raw whole milk samples (A1A1 and A2A2) were collected during the morning milking of selected Friesian cows at local farms (La Cavalleria, Manlleu, Barcelona, Spain and Can Barrina, Santa Cecília de Voltregà, Barcelona, Spain). Cows were carefully selected based on their genetic characteristics, choosing animals with similar age, lactation stage, and number of lactations. Farms were chosen to ensure standardized and consistent animal management and feeding conditions. Samples were immediately cooled to 4 °C and transported to CIRTTA, where fat was separated by centrifugation. The average protein content and pH values of the defatted milk samples were 3.0 ± 0.2 and 3.0 ± 0.3 g/100 mL and 6.63 ± 0.05 and 6.62 ± 0.08 for A1A1 and A2A2 milk, respectively. In vitro digestion was performed following the standardized INFOGEST protocol [15], comprising an oral phase with α-amylase, followed by a 120 min gastric phase with pepsin and a 120 min intestinal phase with pancreatin and bile salts. pH was monitored and adjusted at each phase, incubations were performed using an end-over-end shaker in an oven at 37 °C, and enzyme activities were defined according to supplier specifications and the INFOGEST protocol. Aliquots were collected at three key time points following the addition of the recommended enzyme inhibitors: immediately after completion of the oral phase and addition of the gastric reagents, prior to digestion (0 min); at the end of the gastric phase (120 min); and at the end of the intestinal phase (240 min), corresponding to the initial, gastric (G), and gastrointestinal (GI) fractions, respectively. In parallel, an extended digestion protocol was applied to further evaluate peptide release under prolonged enzymatic exposure, in which the duration of both the gastric and intestinal phases was increased to 240 min each. Aliquots were collected at the corresponding time points for each phase. All collected fractions from the standardized and extended INFOGEST protocols were processed in triplicate by SPE prior to analysis.

An Oasis HLB SPE micro-elution plate was used for clean-up and preconcentration of peptides, including β-CMs, from milk hydrolysates. Following the manufacturer’s recommendations, the SPE plate was initially conditioned with 200 µL of 70% (*v*/*v*) ACN, followed by 200 µL of 0.1% (*v*/*v*) TFA. Next, 500 µL of sample, prefiltered through a 0.22 µm pore nylon filter (Macherey-Nagel, Düren, Germany), was loaded onto the plate. The plate was subsequently washed with 800 µL of 0.1% (*v*/*v*) TFA and 200 µL of water, followed by elution with 50 µL of 70% (*v*/*v*) ACN. The eluate was evaporated using a SpeedVac™ system, and the residue was reconstituted in 50 µL of water, yielding a concentrated sample ready for CE-MS analysis.

#### 2.2.2. CE-MS

Stock solutions of β-CM-5 and β-CM-7 standards were separately prepared at 1000 mg/L in water, then aliquoted and stored in the freezer at −20 °C. Aliquots were defrosted before use, and working standard solutions were prepared by dilution in water.

All CE-MS and CE-MS/MS experiments were performed using a 7100 CE system coupled to a 6546 LC/QTOF mass spectrometer with a triple tube electrospray ionization (ESI) sheath flow interface (Agilent Technologies, Waldbronn, Germany). The sheath liquid was a mixture of propan-2-ol and water (60:40 *v*/*v*) with 0.05% (*v*/*v*) HFor. This solution was delivered at a flow rate of 3.3 µL/min using a KD Scientific 100 series infusion pump (Holliston, MA, USA). Both the sheath liquid and the BGE were degassed for 10 min using ultrasonic agitation before use. CE and QTOF mass spectrometer control, data acquisition, and processing were performed with MassHunter software (version B.10.0, Agilent Technologies). The mass spectrometer was operated in positive ionization mode, and the parameters under optimized conditions were as follows: capillary voltage, 4000 V; drying gas temperature, 200 °C; drying gas flow rate, 4 L/min; nebulizer gas pressure, 7 psig; fragmentor voltage, 215 V; skimmer voltage, 60 V; and OCT 1 RF Vpp, 300 V. MS data were collected in full-scan profile mode at a rate of 1 spectrum/s over the 100–1250 *m*/*z* range. A targeted MS/MS acquisition method was used to confirm peptide identity. Precursor ions were selected according to their theoretical *m*/*z* values (580.2766 for β-CM-5 and 790.4134 for β-CM-7) using a narrow isolation window of approximately 1.3 *m*/*z*, with the precursor charge state fixed at 1. Two collision energies (15 and 30 eV) were evaluated, and 30 eV was selected as it generated the most information-rich fragmentation pattern. MS and MS/MS data were acquired in high resolution (4 GHz). All electrophoretic separations were performed at 25 °C, in 72 cm total length × 75 µm internal diameter (i.d.) fused silica capillaries (Polymicro Technologies, Phoenix, AZ, USA). New capillaries were conditioned disconnected from the CE-MS interface to avoid contamination of the mass spectrometer by flushing at 930 mbar with 1 M NaOH (20 min), water (20 min), and a BGE at pH 2.3 composed of 50 mM HFor and 50 mM HAc (20 min). The conditioned capillary was then installed in the CE-MS interface and equilibrated by applying the separation voltage of 25 kV at positive polarity (cathode in the outlet). Samples were hydrodynamically injected at 50 mbar for 10 s before separation. Between runs, capillaries were conditioned by flushing at 930 mbar with water (3 min) and BGE (3 min). Masshunter software (Agilent Technologies, version B.10.0) was used for instrument control and data analysis.

The CE-MS method was validated using β-CM-5 and β-CM-7 standards through the evaluation of key performance parameters, including linearity, LOD, LOQ, and repeatability, in accordance with established validation principles [29]. Extracted ion electropherograms (EIEs) were obtained for each peptide, smoothed, and automatically integrated with default parameters to obtain migration times and peak areas. Linearity was investigated across the concentration range of 0.05–250 mg/L, and repeatability was determined at 50 mg/L (n = 3) as the relative standard deviation (%RSD) of peak areas and migration times of both peptides. The LODs were established experimentally from the analysis of the most diluted standard solutions of the linearity study until both peptides could no longer be detected (signal-to-noise ratio, S/N < 3). The LOQs were defined as the lowest concentrations within the linear range (S/N > 10). For quantification of β-CM-7 in milk hydrolysates, calibration was performed using both external calibration and standard addition calibration methods to evaluate potential matrix effects on quantification accuracy. External calibration was performed in triplicate at seven concentration levels across the entire linearity range (0.25 to 100 mg/L of β-CM-7). Standard addition calibration was conducted in triplicate by spiking B1-A1A1-GI-120 sample at four different concentration levels (21, 38, 54, and 67 mg/L of β-CM-7).

## 3. Results

### 3.1. Method Development

In general, CE-MS analysis of peptides in positive ESI mode using sheath flow interfaces employs acidic, volatile, and low ionic strength BGEs together with aqueous-organic sheath liquids (typically propan-2-ol or methanol), as these conditions enable appropriate separations while promoting efficient peptide ionization and maximizing sensitivity [21,22,28,30,31,32]. In our previous work on peptide hormones [30], opioid peptides [31], tryptic digests [32], and hypoallergenic milk hydrolysates [28], a BGE composed of 50 mM HAc:50 mM HFor (pH ~2.3), combined with a sheath liquid consisting of 60:40 (*v*/*v*) propan-2-ol/water containing 0.05% (*v*/*v*) HFor, provided excellent analytical performance. These conditions were applied here for the analysis of β-CM-5 and β-CM-7 standard mixtures.

**Figure 1:**
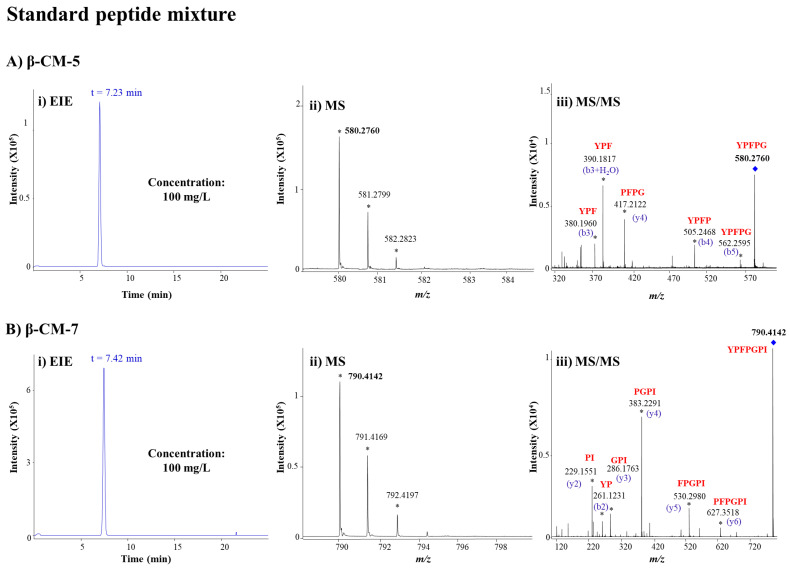
(**i**) EIEs, (**ii**) MS spectra, and (**iii**) MS/MS spectra by CE-MS/MS for (**A**) β-CM-5 and (**B**) β-CM-7 in a 100 mg/L standard mixture. The separation was performed using a fused silica capillary (72 cm total length × 75 µm i.d.), a BGE of 50 mM formic acid and 50 mM acetic acid (pH 2.3), injecting samples hydrodynamically at 50 mbar for 10 s, and applying 25 kV at positive polarity. EIEs were generated by extracting the theoretical monoisotopic [M + H]^+^ ions at *m*/*z* 580.2766 for β-CM-5 and *m*/*z* 790.4134 for β-CM-7. Mass errors in monoisotopic *m*/*z* values for [M + H]^+^ ions of β-CM-5 and β-CM-7 were 1 ppm. Relevant ions in the MS and MS/MS spectra are indicated with an asterisk, and a blue rhombus indicates the precursor ion in the MS/MS spectra.

Figure 1A,B(i) show the EIEs obtained for a 100 mg/L mixture of both peptides. As observed, baseline separation was not achieved at this pH. This outcome was expected given their minimal differences in charge-to-radius ratio and therefore in their electrophoretic mobility [30]. Under the acidic conditions required for optimal sensitivity in positive ESI, they cannot be fully resolved as a function of pH by electrophoretic means alone. However, the peptides were readily distinguished by their different M_r_ values, yielding singly charged protonated ions ([M + H]^+^) at distinct *m*/*z* values, which were resolved with high accuracy using the QTOF mass spectrometer (Figure 1A,B(ii)). Moreover, peptide identity was further confirmed by characteristic MS/MS fragment ions (Figure 1A,B(iii)).

As summarized in Table 1, the CE-MS method demonstrated wide linearity ranges, excellent LODs, and very good repeatability in migration times and peak areas. Linearity was exceptional, with R^2^ values of 0.9991 (0.25–100 mg/L) for β-CM-5 and 0.9976 (0.5–100 mg/L) for β-CM-7, covering the LOQ and the upper limit of linearity. The LODs were 0.10 mg/L for β-CM-5 and 0.25 mg/L for β-CM-7. Migration time and peak area repeatabilities, expressed as %RSD, were also excellent (<0.18% and <1.53%, respectively), indicating the method’s suitability for β-CM analysis in milk hydrolysates generated using the INFOGEST protocol. These figures of merit are consistent with our previous CE-MS studies on peptide hormones and opioid peptides [30,31]. However, given the complexity of the milk hydrolysate matrix and the expected low abundance of β-CMs, sample clean-up and preconcentration using SPE were required. Polymeric reversed-phase HLB microcartridges in micro-elution plate format were selected due to the extensive applicability of HLB SPE in peptide analysis, broad retention range (polar to moderately nonpolar peptides), high recoveries, robustness, and minimal sample and solvents requirements at microscale [33].

### 3.2. Application to Milk Hydrolysates

**Figure 2:**
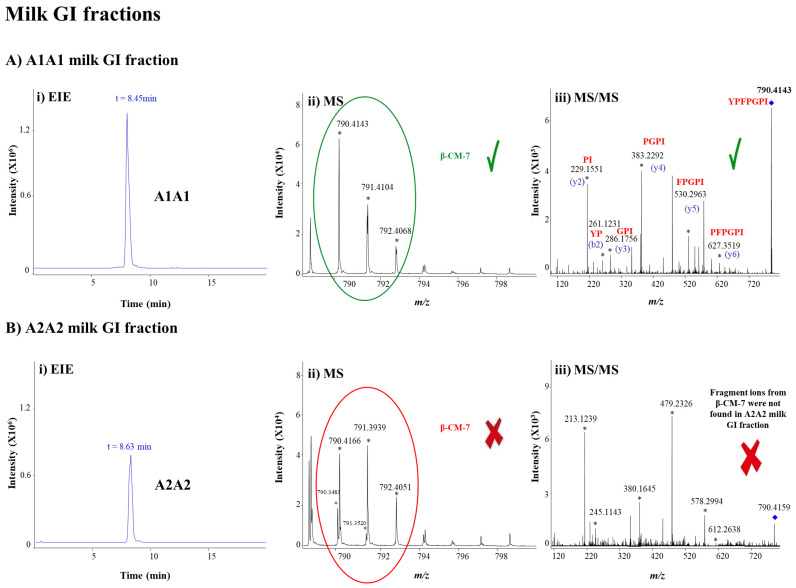
(**i**) EIEs, (**ii**) MS spectra, and (**iii**) MS/MS spectra by CE-MS/MS corresponding to the peak indicative of the potential presence of β-CM-7 in GI fractions from (**A**) A1A1 and (**B**) A2A2 milk samples digested using the standardized INFOGEST protocol. Green (*✓*) and red (X) symbols denote confirmation or exclusion, respectively, of β-CM-7. β-CM-5 was not detected in any of the analyzed samples (no peaks were observed in the corresponding EIEs, Supplementary Figure S1). EIEs for β-CM-7 were generated by extracting the theoretical monoisotopic [M + H]^+^ ion at *m*/*z* 790.4134. Relevant ions in the MS and MS/MS spectra are indicated with an asterisk, and a blue rhombus indicates the precursor ion in the MS/MS spectra.

Application of the CE-MS method to analyze A1A1 and A2A2 hydrolysates from different milk samples at different digestion steps and times using the standardized INFOGEST protocol yielded significant findings. β-CMs were not detected in the initial and G digestion fractions of the A1A1 and A2A2 milk samples. In the remaining GI digestion fraction, as illustrated in Figure 2A, β-CM-7 was clearly identified in A1A1 hydrolysates based on its migration time (Figure 2A(i)), experimental M_r_ (Figure 2A(ii)), and characteristic MS/MS fragment ions (Figure 2A(iii)), consistent with the β-CM-7 standard (Figure 1B(i–iii)).

In contrast, β-CM-5 was not detected in this GI fraction (Supplementary Figure S1), suggesting that, if present, its concentration fell below the LOD (0.10 mg/L). With respect to A2A2 hydrolysates, as shown for the GI digestion fraction in Figure 2B(i), a peak potentially corresponding to β-CM-7 was observed. However, both the relative abundance in the isotopic distribution of the [M + H]^+^ ion (Figure 2B(ii)) and the MS/MS fragmentation pattern (Figure 2B (iii)) differed from those obtained for β-CM-7 in A1A1 hydrolysates corresponding to the GI fraction (Figure 2A(ii,iii)) and from the standard (Figure 1A(ii,iii)). This signal likely originated from another isobaric peptide or small M_r_ constituent generated during digestion of A2A2 milk. As expected, β-CM-5 was not detected in this GI fraction either. These observations, consistent across samples from three independent A1A1 and A2A2 cows, support the hypothesis that GI digestion of β-CN-A1 preferentially yields β-CM-7 rather than β-CM-5, whereas β-CN-A2 does not readily generate neither β-CM-7 nor β-CM-5. This aligns with previous reports showing that β-CM-7 is predominantly released from β-CN-A1 and that β-CN A2 does not produce β-CM-7, suggesting A2 milk may be a preferable option for β-CM-sensitive individuals [3,4,5,6,7].

To further investigate peptide release and assess whether digestion time could influence the generation of these peptides, an extended INFOGEST digestion protocol was applied, in which the duration of both the gastric and intestinal phases was increased to 240 min. Under these conditions, peptide profiles were re-evaluated to determine whether prolonged digestion could promote the release of β-CM-7 or β-CM-5. β-CM-5 was not detected in any G or GI fractions from three independent A1A1 and A2A2 cows, whereas β-CM-7 levels increased following extended enzymatic exposure, as discussed below.

**Figure 3:**
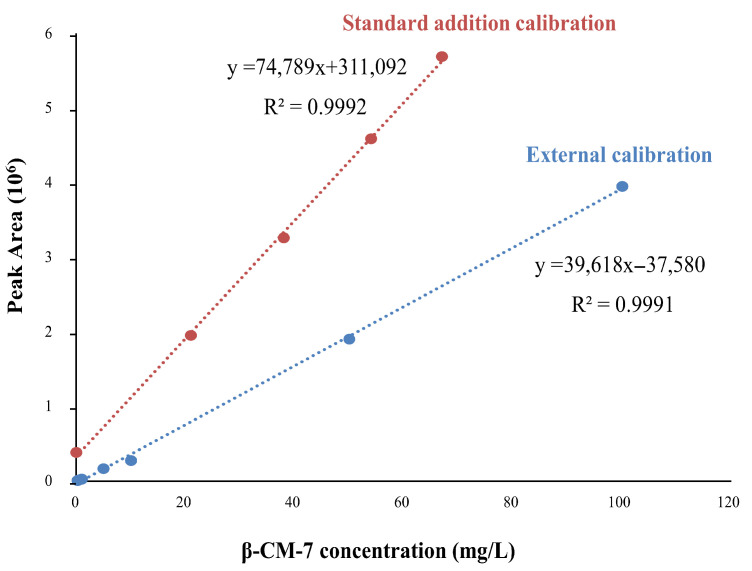
External calibration and standard addition calibration curves for β-CM-7. The GI fraction from B1-A1A1 milk sample digested using the standardized INFOGEST protocol was quantified by standard addition.

Before quantifying β-CM-7 in GI fractions from A1A1 milk, SPE recovery was evaluated by spiking GI fractions obtained using the standardized INFOGEST protocol before and after SPE pretreatment, yielding a recovery of 90%. Table 2 shows β-CM-7 concentrations in GI fractions from three A1A1 milk samples under standardized and extended INFOGEST protocols, quantified using the external calibration curve shown in Figure 3.

β-CM-7 was consistently quantified, with concentrations increasing over time (from ~0.98 mg/L under standardized conditions to ~1.4 mg/L under extended conditions, depending on the sample). Statistical analysis in each case, using an unpaired *t*-test (Welch’s correction), indicated significant differences (*p* < 0.02). Matrix effects were evaluated by comparing external and standard addition calibration curves for one GI fraction (B1-A1A1, standardized INFOGEST protocol). As shown in Figure 3, the slopes differed markedly (74,789 L/mg for standard addition vs. 39,618 L/mg for external calibration), indicating significant matrix effects, likely due to the previously described co-migrating isobaric impurity. This finding suggested that accurate quantification required standard addition calibration for each individual sample. In this particular sample (B1-A1A1 under standardized INFOGEST protocol), β-CM-7 was quantified at 0.42 mg/L using standard addition calibration, compared with 0.98 mg/L using external calibration (Table 2), highlighting the superior accuracy of standard addition in this complex milk hydrolysate matrix, despite prior SPE pretreatment. Similar matrix effects are expected for the GI fractions obtained from the other two A1A1 milk samples under the standardized INFOGEST protocol, as they were prepared under identical conditions from comparable sources and exhibited similar total ion electropherograms, EIEs, MS and MS/MS spectra for β-CM-7. This observation suggests that matrix-matched calibration (e.g., using an A2A2 GI fraction digested under the same conditions as a blank matrix) may represent a straightforward and suitable alternative for accurate quantification.

## 4. Discussion

Previously reported concentrations of β-CM-7 in in vitro digestion studies vary widely depending on the milk or dairy product type, the processing conditions, the digestion protocol, and the analytical technique employed, as reviewed by A. Summer et al. [10]. It should be noted that these in vitro digestion approaches do not account for gastrointestinal absorption, systemic metabolism, or receptor interactions in vivo. Therefore, the β-CM-7 concentrations measured cannot be directly extrapolated to biological effects or human health outcomes. The concentrations obtained in the present study fall within the range reported by A. Cieslinska et al. for A1A1 milk hydrolysates (β-CM-7: 0.373–0.682 mg/L) determined by ELISA [34] and are substantially higher than those described by D. D. Nguyen et al. for heat-treated A1A1 milk hydrolysates (0.127–0.198 mg/L) analyzed by UHPLC-MS/MS [19]. Importantly, neither of these two studies applied a standardized digestion protocol, and different analytical techniques were used. Although the standardized INFOGEST protocol has been widely adopted for the in vitro digestion of dairy proteins, only a limited number of studies have specifically investigated the release of β-CMs under the standardized INFOGEST protocol. Most of the available literature on β-CMs relies on non-standardized digestion conditions or alternative in vitro approaches [10]. This may explain why some authors have reported the detection of β-CM-5 [4,14], which, under the standardized and extended INFOGEST conditions of our study, if present, is found at concentrations below the LOD of the established CE-MS method. The absence of β-CM-5 may reflect alternative, non-exclusive explanations, including limited formation, rapid degradation by intestinal enzymes, or lower stability compared to β-CM-7. Future studies should include a larger and more diverse set of A1 and A2 milk samples to further validate and generalize these findings. This methodological heterogeneity highlights the importance of implementing the standardized INFOGEST protocol and the added value of complementary CE-MS validation to improve comparability, analytical robustness, and reliability in β-CMs research. CE-MS provides an MS-based microseparation alternative to LC-MS for the determination of β-CMs in bovine milk digests, offering several well-established advantages, including a complementary separation mechanism, low consumption of samples, reagents, and solvents, and short analysis times [21,22,23,24]. The observed matrix effects represent a limitation that necessitates the use of standard addition or matrix-matched calibration for accurate quantification. However, this requirement does not outweigh the analytical benefits of CE-MS, which deserves to be directly compared with LC-MS in future studies.

## 5. Conclusions

In this study, a rapid, selective, and sensitive CE-MS method was developed for the accurate identification and quantification of β-CM-7 and β-CM-5 in milk hydrolysates generated after in vitro GI digestion using the standardized INFOGEST protocol. The method showed satisfactory linearity (0.5–100 mg/L β-CM-7 and 0.25–100 mg/L β-CM-5), LODs (0.25 mg/L β-CM-7 and 0.10 mg/L β-CM-5), and LOQs (0.5 mg/L β-CM-7 and 0.25 mg/L β-CM-5). Migration time and peak area repeatabilities were also acceptable (<0.18 and <1.53%RSD, respectively), while MS/MS characterization ensured confident peptide confirmation. When applied to A1A1 and A2A2 milk hydrolysates, clean-up and preconcentration using HLB SPE provided excellent peptide recoveries (90%). β-CM-7 was consistently detected and quantified in GI fractions from A1A1 milk, whereas β-CM-5 was not detected, even under extended GI digestion time. In contrast, neither β-CM-7 nor β-CM-5 was detected in GI fractions from A2A2 milk, confirming the resistance of β-CN-A2 to digestion under the applied conditions. The concentrations of β-CM-7 measured in GI fractions from A1A1 milk using external calibration were consistent with values previously reported in the literature (<1 mg/L under standardized conditions). However, for more accurate quantification, the use of standard addition or matrix-matched calibration is recommended, given the presence of matrix effects. Overall, these results confirm both the reliability of the developed CE-MS method and the strong influence of β-CN genetic variants on β-CM-7 release, which depends on the duration of the intestinal phase. Future studies should include a larger and more diverse set of A1 and A2 milk samples, the exploration of novel sample preparation approaches to minimize matrix effects, and direct comparison with LC-MS. The proposed CE-MS method thus constitutes a valuable analytical tool for reliable β-CM profiling in bovine milk from different genotypes following digestion under the standardized INFOGEST protocol, supporting quality control and the safety assessment of A2 milk and derived dairy products. It also provides a robust starting point for in vivo studies addressing potential health implications and can be extended to investigations of other bioactive peptides released from milk proteins.

## Figures and Tables

**Table 1:** Linearity, limits of detection (LOD), limits of quantification (LOQ), and repeatability of the CE-MS method for β-CM-5 and β-CM-7.

Quality Parameter	β-CM-5	β-CM-7
Linearity range (mg/L)	0.25–100	0.5–100
R^2^	0.9991	0.9976
LOD (mg/L)	0.10	0.25
LOQ (mg/L)	0.25	0.5
Repeatability migration time %RSD (n = 3)	0.18	0.11
Repeatability peak area %RSD (n = 3)	0.32	1.53

**Table 2:** β-CM-7 concentrations (average value ± standard deviation, n = 3) in GI fractions from three A1A1 milk samples digested using standardized and extended INFOGEST protocols, quantified using the external calibration curve shown in Figure 3. β-CM-5 was not detected in A1A1 milk digestion fractions, and no β-CMs were detected in A2A2 milk digestion fractions.

Sample Codes	β-CM-7 (mg/L) Standardized INFOGEST: G (120 min) + I (120 min)	β-CM-7 (mg/L) Extended INFOGEST: G (240 min) + I (240 min)
B1-A1A1	0.98 ± 0.04	1.36 ± 0.07
B2-A1A1	0.98 ± 0.08	1.46 ± 0.09
C-A1A1	0.98 ± 0.02	1.37 ± 0.03

## Data Availability

The original contributions presented in this study are included in the article/Supplementary Materials. Further inquiries can be directed to the corresponding author.

## Supplementary Materials

The following supporting information can be downloaded at https://www.mdpi.com/article/10.3390/foods15101776/s1: Figure S1: (i) EIEs and (ii) MS spectra, by CE-MS at the migration time where β-CM-5 was expected to be detected, comigrating with the potential β-CM-7 peak (Figure 2), in GI fractions from (A) A1A1 and (B) A2A2 milk samples digested using the standardized INFOGEST protocol. Red (X) symbols denote that β-CM-5 was not detected in any of the analyzed samples. The ions observed in the mass spectra correspond to other compounds detected at the selected migration time. EIEs for β-CM-5 were generated by extracting the monoisotopic [M + H]^+^ ion at *m*/*z* 580.2766.

## Abbreviations

The following abbreviations are used in this manuscript:

BGE	Background electrolyte
β-CN-A1	β-Casein A1
β-CN-A2	β-Casein A2
β-CM-5	β-casomorphin-5
β-CM-7	β-casomorphin-7
CE-MS	Capillary electrophoresis–mass spectrometry
CE-MS/MS	Capillary electrophoresis–tandem mass spectrometry
EIE	Extracted ion electropherogram
FA	Formic acid
G	Gastric
GI	Gastrointestinal
HAc	Acetic acid
HLB	Hydrophilic lipophilic balance
I	Intestinal
i.d.	Internal diameter
LC-MS	Liquid chromatography–mass spectrometry
LC-MS/MS	Liquid chromatography–tandem mass spectrometry
LOD	Limit of detection
LOQ	Limit of quantification
M_r_	Molecular mass
MS	Mass spectrometry
MS/MS	Tandem mass spectrometry
QTOF	Quadrupole time-of-flight
R^2^	Coefficient of determination
RSD	Relative standard deviation
SPE	Solid-phase extraction
TFA	Trifluoroacetic acid

## References

[1] De Jong N., Visser S., Olieman C. (1993). Determination of Milk Proteins by Capillary Electrophoresis. J. Chromatogr. A.

[2] Farrell H.M., Jimenez-Flores R., Bleck G.T., Brown E.M., Butler J.E., Creamer L.K., Hicks C.L., Hollar C.M., Ng-Kwai-Hang K.F., Swaisgood H.E. (2004). Nomenclature of the Proteins of Cows’ Milk—Sixth Revision. J. Dairy Sci..

[3] Juan B., Salama A.A.K., Serhan S., Such X., Caja G., Pont L., Benavente F., Guamis B., Trujillo A.-J. (2024). β-Casein: Type A1 and A2. Casein.

[4] Cieślińska A., Fiedorowicz E., Rozmus D., Sienkiewicz-Szłapka E., Jarmołowska B., Kamiński S. (2022). Does a Little Difference Make a Big Difference? Bovine β-Casein A1 and A2 Variants and Human Health—An Update. Int. J. Mol. Sci..

[5] Pal S., Woodford K., Kukuljan S., Ho S. (2015). Milk Intolerance, Beta-Casein and Lactose. Nutrients.

[6] Ji D., Ma J., Dai J., Xu M., Harris P.W.R., Brimble M.A., Agyei D. (2022). Anticholinesterase Inhibition, Drug-Likeness Assessment, and Molecular Docking Evaluation of Milk Protein-Derived Opioid Peptides for the Control of Alzheimer’s Disease. Dairy.

[7] Barnett M.P.G., Mcnabb W.C., Roy N.C., Woodford K.B., Clarke A.J. (2014). Dietary A1 β-Casein Affects Gastrointestinal Transit Time, Dipeptidyl Peptidase-4 Activity, and Inflammatory Status Relative to A2 β-Casein in Wistar Rats. Int. J. Food Sci. Nutr..

[8] Kamiński S., Cieślińska A., Kostyra E. (2007). Polymorphism of Bovine Beta-Casein and Its Potential Effect on Human Health. J. Appl. Genet..

[9] Nogueira A.C.R., Ferreira T.d.O., Ribeiro F.B., Amorim K.A., Bastos S.C., da Cruz A.G., Rodrigues J.F. (2025). A2 Milk: Perceptions, Purchase Intentions and Reports during Consumption. Int. J. Dairy Technol..

[10] Summer A., Di Frangia F., Ajmone Marsan P., De Noni I., Malacarne M. (2020). Occurrence, Biological Properties and Potential Effects on Human Health of β-Casomorphin 7: Current Knowledge and Concerns. Crit. Rev. Food Sci. Nutr..

[11] Daniloski D., Cunha N.M.D., McCarthy N.A., O’Callaghan T.F., McParland S., Vasiljevic T. (2021). Health-Related Outcomes of Genetic Polymorphism of Bovine β-Casein Variants: A Systematic Review of Randomised Controlled Trials. Trends Food Sci. Technol..

[12] Edwards T.S., Dawson K.L., Keenan J.I., Day A.S. (2021). A Simple Method to Generate β-Casomorphin-7 by in Vitro Digestion of Casein from Bovine Milk. J. Funct. Foods.

[13] Asledottir T., Le T.T., Poulsen N.A., Devold T.G., Larsen L.B., Vegarud G.E. (2018). Release of β-Casomorphin-7 from Bovine Milk of Different β-Casein Variants after Ex Vivo Gastrointestinal Digestion. Int. Dairy J..

[14] De Noni I., Cattaneo S. (2010). Occurrence of β-Casomorphins 5 and 7 in Commercial Dairy Products and in Their Digests Following in Vitro Simulated Gastro-Intestinal Digestion. Food Chem..

[15] Brodkorb A., Egger L., Alminger M., Alvito P., Assunção R., Ballance S., Bohn T., Bourlieu-Lacanal C., Boutrou R., Carrière F. (2019). INFOGEST Static in Vitro Simulation of Gastrointestinal Food Digestion. Nat. Protoc..

[16] Nguyen D.D., Busetti F., Johnson S.K., Solah V.A. (2015). Identification and Quantification of Native Beta-Casomorphins in Australian Milk by LC–MS/MS and LC–HRMS. J. Food Compos. Anal..

[17] Nguyen D.D., Busetti F., Johnson S.K., Solah V.A. (2018). Evaluation of a Commercial Sandwich Enzyme-Linked Immunosorbent Assay for the Quantification of Beta-Casomorphin 7 in Yogurt Using Solid-Phase Extraction Coupled to Liquid Chromatography-Tandem Mass Spectrometry as the “Gold Standard” Method. J. AOAC Int..

[18] Nguyen D.D., Solah V.A., Johnson S.K., Nguyen H.A., Nguyen T.L.D., Tran T.L.H., Mai T.K., Busetti F. (2019). Identification and Quantification of Beta-Casomorphin Peptides Naturally Yielded in Raw Milk by Liquid Chromatography-Tandem Mass Spectrometry. LWT.

[19] Nguyen D.D., Busetti F., Smolenski G., Johnson S.K., Solah V.A. (2021). Release of Beta-Casomorphins during in-Vitro Gastrointestinal Digestion of Reconstituted Milk after Heat Treatment. LWT.

[20] Ghafoori Z., Tehrani T., Pont L., Benavente F. (2022). Separation and Characterization of Bovine Milk Proteins by Capillary Electrophoresis-mass Spectrometry. J. Sep. Sci..

[21] Lauer H.H., Rozing G.P. (2014). High Performance Capillary Electrophoresis.

[22] Rozing G., Brunnert H., Aldridge S., Wenz C. (2019). CE/MS Principles and Practices.

[23] Kašička V. (2024). Recent Developments in Capillary and Microchip Electroseparations of Peptides (2021–Mid-2023). Electrophoresis.

[24] Kašička V. (2026). Recent Developments in Capillary and Microchip Electroseparations of Peptides (2023–Mid 2025). Electrophoresis.

[25] Patel V.D., Shamsi S.A., Sutherland K. (2021). Capillary Electromigration Techniques Coupled to Mass Spectrometry: Applications to Food Analysis. Trends Anal. Chem..

[26] Castro-Puyana M., Marina M.L. (2023). Capillary Electrophoresis-Mass Spectrometry-based approaches for food analysis and food metabolomics. Curr. Opin. Food Sci..

[27] Semail N.-F., Yahaya N., Mohamed A.H., Chen D.D.Y., Zain N.N.M. (2025). Advances and Applications of Capillary Electrophoresis Mass Spectrometry in Food Analysis: Strategies for Online and Offline Preconcentration. Electrophoresis.

[28] Català-Clariana S., Benavente F., Giménez E., Barbosa J., Sanz-Nebot V. (2013). Identification of Bioactive Peptides in Hypoallergenic Infant Milk Formulas by CE-TOF-MS Assisted by Semiempirical Model of Electromigration Behavior. Electrophoresis.

[29] Cantwell H. (2025). Eurachem Guide: The Fitness for Purpose of Analytical Methods—A Laboratory Guide to Method Validation and Related Topics.

[30] Benavente F., Balaguer E., Barbosa J., Sanz-Nebot V. (2006). Modelling Migration Behavior of Peptide Hormones in Capillary Electrophoresis-Electrospray Mass Spectrometry. J. Chromatogr. A.

[31] Medina-Casanellas S., Benavente F., Barbosa J., Sanz-Nebot V. (2011). Transient Isotachophoresis in On-Line Solid Phase Extraction Capillary Electrophoresis Time-of-Flight-Mass Spectrometry for Peptide Analysis in Human Plasma. Electrophoresis.

[32] Wenz C., Barbas C., Lõpez-Gonzálvez Á., Garcia A., Benavente F., Sanz-Nebot V., Blanc T., Freckleton G., Britz-McKibbin P., Shanmuganathan M. (2015). Interlaboratory Study to Evaluate the Robustness of Capillary Electrophoresis-Mass Spectrometry for Peptide Mapping. J. Sep. Sci..

[33] Giménez E., Pont L., Benavente F. (2025). Sample Preparation Methods in Bottom-up Proteomics. Comprehensive Sampling and Sample Preparation.

[34] Cieślińska A., Kostyra E., Kostyra H., Oleński K., Fiedorowicz E., Kamiński S. (2012). Milk from cows of different β-casein genotypes as a source of β-casomorphin-7. Int. J. Food Sci. Nutr..

