# Supplementary figures and images for: Differential Release of β-Casomorphins from A1 and A2 Milk During Standardized Gastrointestinal Digestion Quantified by CE–MS
Source: Foods. 2026 May 18;15(10):1776. doi: 10.3390/foods15101776 (PMC13205146; doi:10.3390/foods15101776)

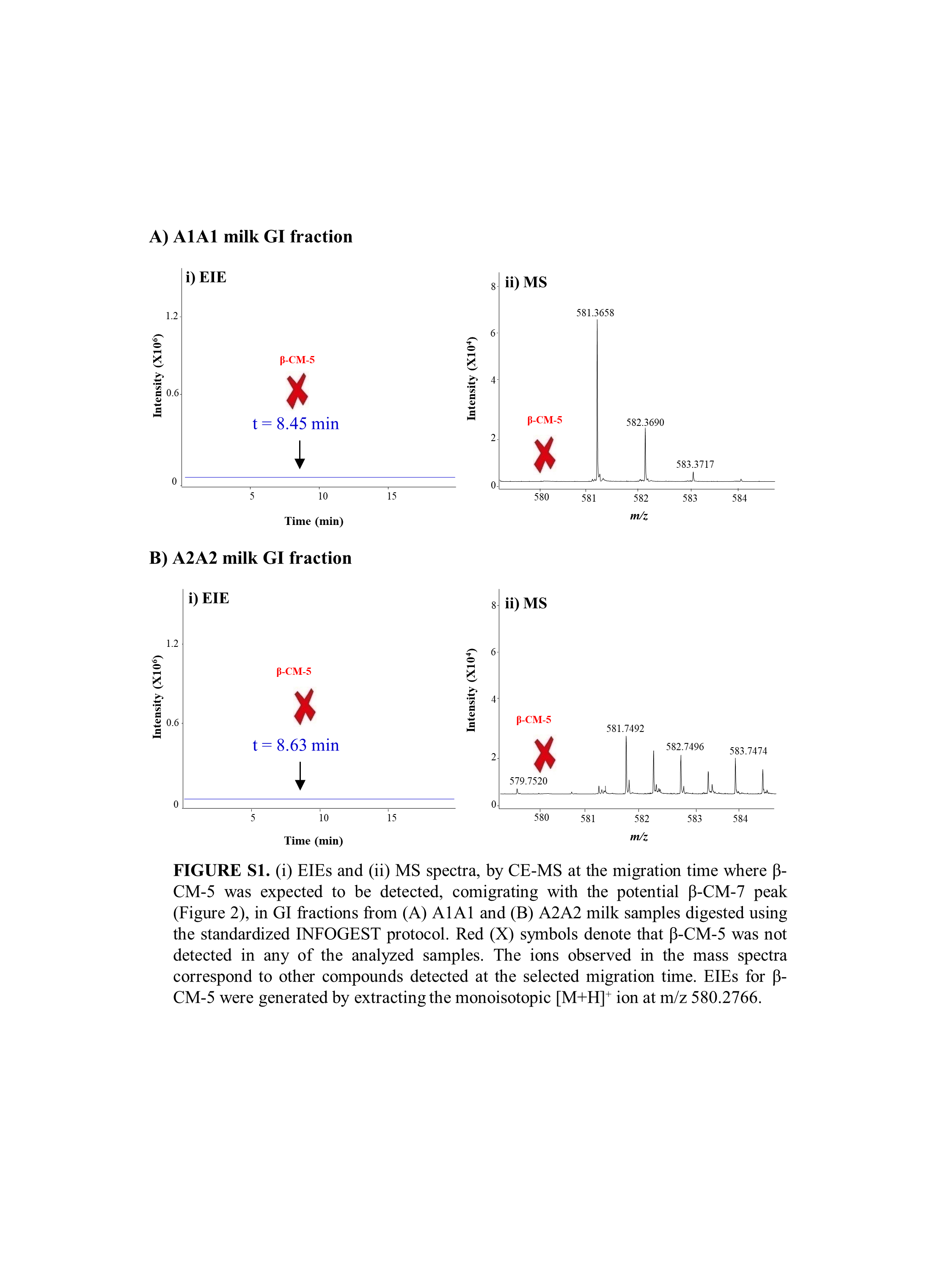

Supplement: Supplementary file 1 [file foods-15-01776-s001.zip › foods-4283793-supplementary.tif]
